# Versatile Tunable Terahertz Absorption Device Based on Bulk Dirac Semimetals and Graphene

**DOI:** 10.3390/molecules30050999

**Published:** 2025-02-21

**Authors:** Jie Zhou, Xin Sun, Jun Xu, Shiyue Wu, Kaili Jin, Yongjian Tang, Zao Yi, Yougen Yi

**Affiliations:** 1 School of Electronic Information and Electrical Engineering, Chengdu University, Chengdu 610100, China; zhoujie_cc@163.com; 2 School of Materials Science and Engineering, Tsinghua University, Beijing 100084, China; 3 College of Urban Construction, Zhejiang Shuren University, Hangzhou 310015, China; 4 Institute of Guizhou Aerospace Measuring and Testing Technology, Guiyang 550009, China; wushiy@163.com (S.W.); jinkl845@163.com (K.J.); 5 Joint Laboratory for Extreme Conditions Matter Properties, Southwest University of Science and Technology, Mianyang 621010, China; tangyongjian2000@sina.com (Y.T.); yizaomy@swust.edu.cn (Z.Y.); 6 College of Physics, Central South University, Changsha 410083, China; yougenyi@csu.edu.cn

**Keywords:** absorber, metamaterial, Dirac semimetal, graphene, terahertz, wideband absorption

## Abstract

We employed the CST Microwave Studio software 2020 and the FDID algorithm for simulation. We have designed a terahertz broadband absorber based on Dirac semimetals and graphene, achieving continuous broadband absorption with a rate exceeding 80% over the range from 7.6776 to 9.172 THz. This broadband absorber features two independent tuning modes, utilizing graphene and Dirac semimetals, and exhibits strong electromagnetic adaptability. Furthermore, we conducted an in-depth analysis of the physical mechanisms underlying the high absorption in these absorbers using impedance matching theory and localized surface plasmon resonance (LSPR) theory. Variations in the dielectric constants of different dielectric layers and the relaxation time of graphene can also modulate the absorption rate. In summary, our proposed terahertz broadband absorber, employing two distinct tunable materials, enhances the device’s flexibility and environmental adaptability, offering promising prospects for wideband absorption applications.

## 1. Introduction

Currently, there remains substantial room for advancement in the development and utilization of the terahertz frequency range (0.1–10 THz), often referred to as the “terahertz gap” [1,2]. Researchers have recognized the significance of this range and the vast potential it holds. Terahertz (THz) waves are poised to become the communication band for next-generation technologies, including 6G. They hold the potential to advance various fields such as defense, space exploration, biomedical applications, and economic development, offering new impetus for the progress of human civilization [3,4,5]. The availability of sophisticated electromagnetic sensors is a prerequisite for the effective exploitation of specific frequency ranges and the advancement of related technologies. However, the development and utilization of electromagnetic waves within the frequency range known as the “terahertz gap” (10^11^–10^13^ Hz), characterized by its wide bandwidth, low energy, strong penetration, and high resolution, have long remained underexplored [6,7,8]. A critical factor for the advancement of electromagnetic wave technologies is the availability of functional devices capable of responding to specific frequency bands. Among these, electromagnetic wave absorption is particularly significant, as it forms the foundation for effective utilization of such waves [9,10].

The terahertz gap lies at the intersection of electronics and photonics in the electromagnetic spectrum, making it challenging for natural materials to exhibit robust responses to terahertz waves. Consequently, the exploitation of this frequency range necessitates the development of materials with specialized electromagnetic properties.

Metamaterials, with their ability to be flexibly designed due to their periodic atomic structures, offer extraordinary electromagnetic characteristics not found in traditional materials [11,12,13]. The flexibility of metamaterials has led to the design of various electromagnetic absorbers, addressing the limitations of natural materials and potentially bridging the existing terahertz gap [14]. Since Landy et al. introduced the first metamaterial absorber in 2008, there has been considerable interest in the research and application of metamaterials in the electromagnetic field [15]. This has highlighted the immense potential of metamaterials in electromagnetic wave technologies. In recent years, graphene, noted for its exceptional optoelectronic properties, has garnered significant attention and is often referred to as a “game-changing material” [16,17,18]. Additionally, bulk Dirac semimetals, which represent a three-dimensional counterpart to graphene, have also attracted growing interest and are considered a “three-dimensional graphene” [19,20,21,22]. Hu et al. proposed a dynamic triple adjustable dual frequency metamaterial absorber that can be electrically, thermally, and magnetically controlled [23]. Qi et al. developed a dual-band terahertz broadband absorber using graphene [24].

Building upon the current research on terahertz absorbers based on graphene, Dirac semimetals, and other metamaterials, this study explores a dual-control broadband terahertz absorber combining the strengths of Dirac semimetals and graphene. This device achieves continuous broadband absorption with an efficiency exceeding 80% over the range from 7.6776 to 9.172 THz. Given that both Dirac semimetals and graphene can independently tune the Fermi level, the electromagnetic adaptability of this device is notably robust. We conducted a thorough investigation into the physical mechanisms underlying the high absorption using impedance matching theory and localized surface plasmon resonance (LSPR) theory. The impact of different dielectric layer refractive indices on device performance was also examined. Additionally, we explored the influence of the properties of the two metamaterials on the device. This absorber is expected to have significant applications in terahertz modulation and attenuation.

## 2. Modeling and Structural Parameters of the Micro-Nano Optical Devices

**Figure 1:**
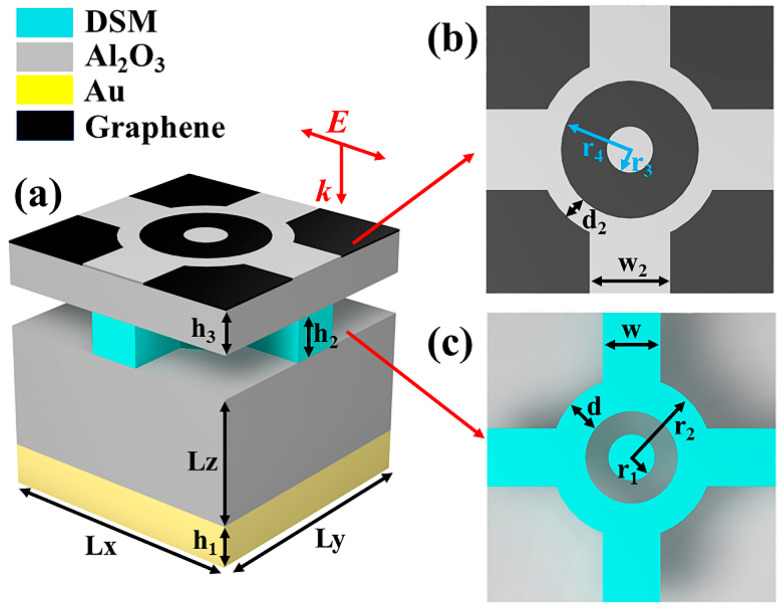
(**a**) Schematic diagram of the 3D structure of the designed wideband absorber. (**b**) Structural diagram of the graphene layer. (**c**) Structural diagram of DSM layer.

The proposed structure of our terahertz absorber is illustrated in Figure 1a, featuring a four-layer configuration. The structure of the top graphene layer is designed to be approximately complementary to that of the intermediate Dirac semimetal (DSM) layer when viewed from the projection plane. The uncovered portions of the graphene layer ensure that electromagnetic waves can penetrate to the DSM layer, thereby eliciting additional electromagnetic responses. The graphene pattern is derived from a difference set operation involving a square, a circle, an annulus, and four rectangles connected to the annulus, with detailed structural parameters provided in Table 1. The conductivity of gold is 4.561 × 10^+007^ S/m, and the dielectric constant of aluminum oxide is 2.28.

The initial Fermi level of the graphene was set to 0.6 eV, with an initial relaxation time of 0.6 ps and a thickness of 1 nm. The initial Fermi level of the Dirac semimetal (DSM) was set to 90 meV. For the simulation, we employed CST software and utilized the FDID algorithm [25,26]. Periodic boundary conditions were applied in the X and Y directions, while open boundary conditions were established in the Z direction, allowing electromagnetic waves to incident normally from the negative *z* axis onto the surface of the device [27,28].

Then, we gave the manufacturing methods in bottom-up order. First, a 0.5 μm gold film was sputtered on the prepared substrate, and then a 12 μm thick alumina dielectric layer was grown by CVD. After that, a layer of Dirac semimetal film was sputtered again, with a thickness of 2 μm, and the desired microstructure was obtained through gluing, development, exposure, etching, and stripping processes. The same process can be followed separately to obtain an alumina dielectric layer and a graphene layer, which can then be covered on top of the Dirac semimetal film. The repeated lithography process finally obtains the array structure of the absorber [29,30].

In this paper, graphene conductivity can be described by the Kubo formula [31,32]:

(1) $$\sigma_{Graphene}=\sigma_{G\_intra}+\sigma_{G\_inter}$$

In the above formula, σ_G_intra_ and σ_G_inter_ are as follows:

(2) $$\sigma_{G\_intra}=\frac{ie^{2}k_{B}T}{\pi \hbar^{2}(\omega +i\tau^{-1})}\left[\frac{E_{F}}{K_{B}T}+2ln(e^{-\frac{E_{F}}{k_{B}T}}+1)\right]$$

(3) $$\sigma_{G\_inter}=\frac{ie^{2}}{4\pi \hbar^{2}}ln\left[\frac{2\left|E_{F}\right|-\hbar (\omega +i\tau^{-1})}{2\left|E_{F}\right|+\hbar (\omega +i\tau^{-1})}\right]$$

Here, *ħ* represents the reduced Planck constant, k_B_ is the Boltzmann constant, e denotes the charge of an electron, and E_F_ and τ refer to the Fermi level and relaxation time of the graphene layer, respectively. ω represents the angular frequency of the incident wave, and T indicates the ambient temperature. Within the frequency range under investigation, the interband conductivity σ_G_intra_ of graphene can be considered negligible (since σ_G_intra_ approaches zero when E_F_ >> kT). Thus, at room temperature (298 K), the conductivity of graphene can be expressed in the Drude form [33]:

(4) $$\sigma \left(\omega \right)=\frac{ie^{2}\left|E_{F}\right|}{\pi \hbar^{2}(\omega +i\tau^{-1})}$$

In the above formula,

(5) $$\tau =\frac{E_{F}v}{ev_{F}^{2}}$$

In Equation (4), v denotes the carrier mobility of the top-layer graphene material, and v_F_ = 10^6^ m/s is the Fermi velocity. Additionally, the dielectric constant of graphene can be expressed as follows [34]:

(6) $$\varepsilon =1+\frac{i\sigma_{Graphene}}{\omega \varepsilon_{0}∆}$$

where ε_0_ is the vacuum dielectric constant, and ∆ represents the thickness of the graphene.

At the same time, the following expression can also be obtained by using the Kubo formula for Dirac semimetals [35]:

(7) $$\sigma_{D\_intra}=\frac{ie^{2}}{\hbar }\frac{gk_{F}}{6\pi^{2}\Omega }\left[1+\frac{\pi^{2}}{3}{\left(\frac{T}{E_{F}}\right)}^{2}\right]$$

(8) $$\sigma_{D\_inter}=\frac{ie^{2}g\omega }{3\pi^{2}\hbar v_{F}}\left[-\frac{\pi i}{2}\frac{G\left(\frac{\hbar \omega }{2}\right)}{4}+\int_{0}^{\infty }\left(\frac{G\left(\varepsilon \right)-G(\hbar \omega /2)}{\hbar^{2}\omega^{2}-4\varepsilon^{2}}\right)\varepsilon d\varepsilon \right]$$

Here, g = 40 is the damping formula, Ω = ℏω/E_F_, and ε_c_ = E_c_/E_F_ (where ε_c_ = 3 represents the cutoff energy) are parameters related to the damping, $G\left(E\right)=sinh(E/T)/\left[\cosh\left(E_{F}/T\right)+cosh(E/T)\right]\;$ [36] is a relevant constant, and k_F_ = E_F_/(ℏv_F_) denotes the Fermi momentum. According to the Random Phase Approximation theory, the real and imaginary parts of the dynamic conductivity under condition T << E_F_ can be expressed as follows [37]:

(9) $$Re\;\sigma_{Dirac}\left(\Omega \right)=\frac{e^{2}}{\hbar }\frac{gk_{F}}{24\pi }\Omega G\left(\frac{\Omega }{2}\right)$$

(10) $$Im\;\sigma_{Dirac}\left(\Omega \right)=\frac{e^{2}}{\hbar }\frac{gk_{F}}{24\pi^{2}}\left\{\frac{4}{\Omega }\left[1+\frac{\pi^{2}}{3}{\left(\frac{T}{E_{F}}\right)}^{2}\right]+8\Omega \int_{0}^{\varepsilon_{c}}\left[\frac{G\left(\varepsilon \right)-G(\frac{\Omega }{2})}{\Omega^{2}-4\varepsilon^{2}}\right]\varepsilon d\varepsilon \right\}$$

Then, based on the two-band model, taking into account interband electron transport, the effective dielectric constant of the DSM can be expressed as follows [38,39]:

(11) $$\varepsilon_{Dirac}=\varepsilon_{b}+\frac{i\sigma_{Dirac}}{\omega \varepsilon_{0}}=\varepsilon_{b}+i\frac{Re(\sigma_{Dirac}\left(\Omega \right))+Im(\sigma_{Dirac}\left(\Omega \right))}{\omega \varepsilon_{0}}$$

where ε_0_ = 1, and ε_b_ = 1.

## 3. Results and Discussion

Through the preceding analysis, we obtained the S parameters. Subsequently, we utilized these S parameters to calculate the absorption rate (A) and transmission rate (T). The absorption rate is expressed as A = 1 − R − T, where R represents the reflectance, given by R =$\;\left|S_{11}\right|$^2^, and the transmission rate is defined as T = $\;\left|S_{21}\right|$^2^ [40,41]. Notably, the thickness of the underlying metallic reflective layer is configured to ensure that terahertz waves do not propagate through the absorber. Thus, the absorption rate can be simplified to A = 1 − R [36,42].

**Figure 2:**
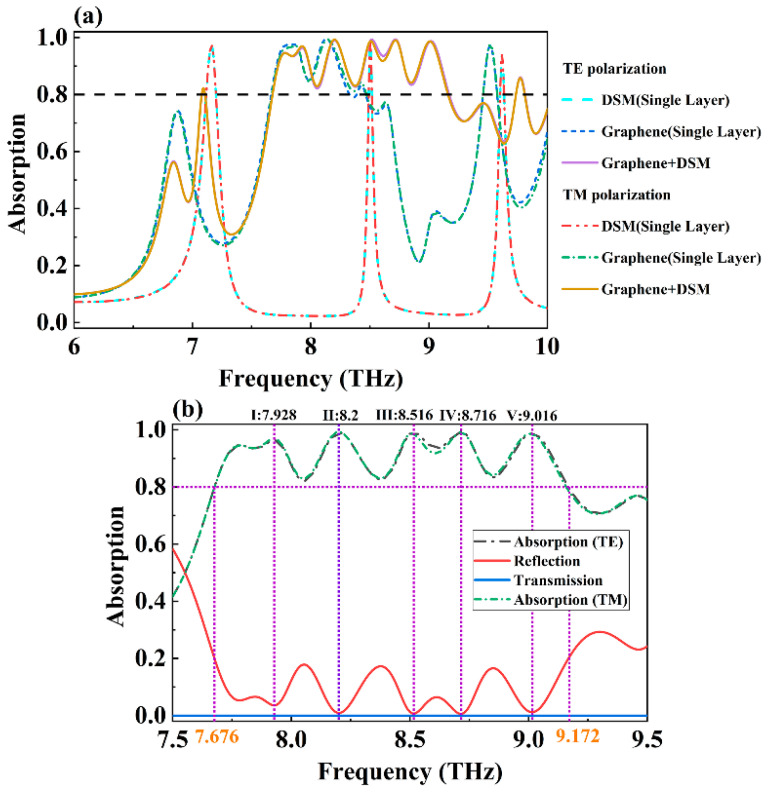
(**a**) Absorption spectra of different structures of absorbers under the incidence of TE and TM electromagnetic waves; (**b**) the absorption, reflection, and transmission spectra of the designed wideband absorber.

Figure 2a presents a comparative analysis of the absorption spectra for different absorber structures within the 6–10 THz frequency range under TE and TM mode electromagnetic wave incidence. The figure includes three absorber configurations: Absorber One, Absorber Two, and an absorber with only the graphene layer, after removing the intermediate Dirac semimetal (DSM) layer. All three absorber structures exhibited perfect rotational symmetry, ensuring that the absorption spectra corresponding to the two modes of incident electromagnetic waves would be identical [43]. When the absorber consisted solely of the graphene layer, it achieved continuous broadband absorption with an efficiency exceeding 80% over the range of 7.668–8.328 THz. This performance was evaluated using the following formula [44]:

(12) $$B_{w}=2\times \frac{\left(f_{max}-f_{min}\right)}{\left(f_{max}+f_{min}\right)}\times 100\%$$

where f_max_ and f_min_ represent the highest and lowest frequencies of the corresponding absorption bands, respectively. The total absorption bandwidth of the device was calculated to be 0.66 THz, with a relative bandwidth (B_w_) of 8.25%. However, the incorporation of graphene with a Dirac semimetal (DSM) significantly enhanced the broadband absorption characteristics of the absorber. For Absorber Two, the frequency range where the absorption efficiency exceeded 80% extended from 7.676 to 9.172 THz, with a total absorption bandwidth of 1.496 THz and a relative bandwidth of 17.76%, centered at 8.424 THz. This represents more than a two-fold improvement in broadband absorption performance compared to the graphene-only absorber. The superior broadband absorption is attributed to the synergistic effect of the top graphene layer and the intermediate DSM layer, with the DSM enhancing the response to electromagnetic waves [45,46]. As depicted in Figure 2b, within the 7.5–9.5 THz range, Absorber Two exhibited five prominent resonant absorption peaks, located at central frequencies of 7.928 THz (Mode I, absorption rate of 96.391%), 8.2 THz (Mode II, absorption rate of 99.05%), 8.516 THz (Mode III, absorption rate of 99.28%), 8.716 THz (Mode IV, absorption rate of 99.37%), and 9.016 THz (Mode V, absorption rate of 98.775%). The excellent broadband absorption characteristics of this absorber suggest its promising applications in terahertz wave modulation and attenuation [47].

**Figure 3:**
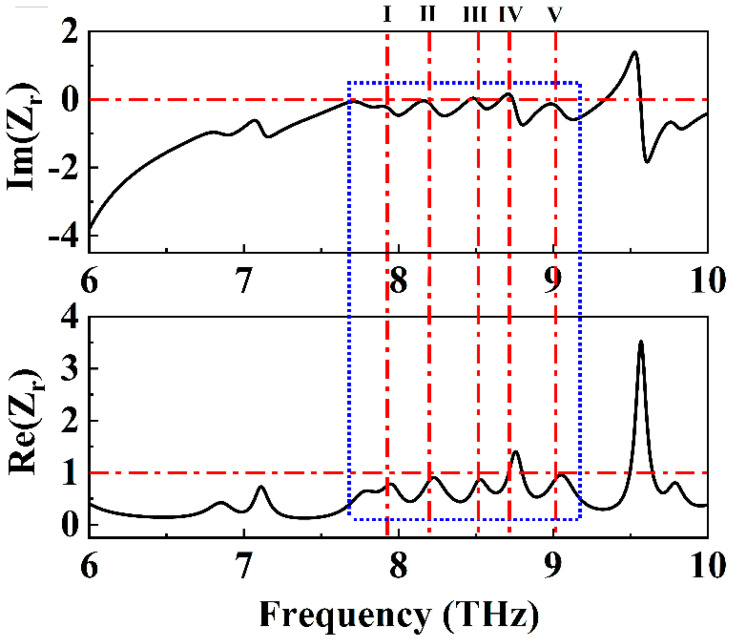
Virtual and real parts of the relative impedance of a wideband absorber in the range of 6–10 THz.

We know that in the terahertz region, impedance matching theory has been used experimentally to achieve broadband absorption in other wavelength ranges, such as the microwave and visible to infrared regions. Therefore, we looked to better explain the absorption effect demonstrated by our above absorption devices. When the equivalent impedance of the absorber matches the impedance of the free space, the relative impedance Z = 1, the device will reach the critical coupling condition, thus showing strong electromagnetic absorption characteristics [48]. We calculated the relative impedance of the absorber in the 6–10 THz range according to Equation (13), with the results shown in Figure 3. The highlighted region in the blue dashed box corresponds to the 7.676–9.172 THz frequency range. It is evident that within the frequency range where the absorber achieved an absorption efficiency greater than 80%, the real part of the relative impedance remained close to 1, exhibiting only minor fluctuations. Similarly, the imaginary part remained near 0 with minimal variation. Throughout the strong absorption band, the relative impedance of the absorber was well matched with the impedance of free space, achieving near-perfect matching at the center frequencies of the five absorption peaks, where Im(Z_r_) approaches 0 and Re(Z_r_) approaches 1 [49]. This impedance matching elucidates the mechanism behind the absorber’s broadband absorption performance from the perspective of impedance matching theory [50,51]:

(13) $$z_{r}=\pm \sqrt{\frac{{\left(1+S_{11}\right)}^{2}-S_{21}^{2}}{{\left(1-S_{11}\right)}^{2}-S_{21}^{2}}}=\frac{z}{\eta }$$

Here, η represents the impedance in free space, z represents the impedance of the absorber, and $z_{r}$ represents the relative impedance of the absorber.

**Figure 4:**
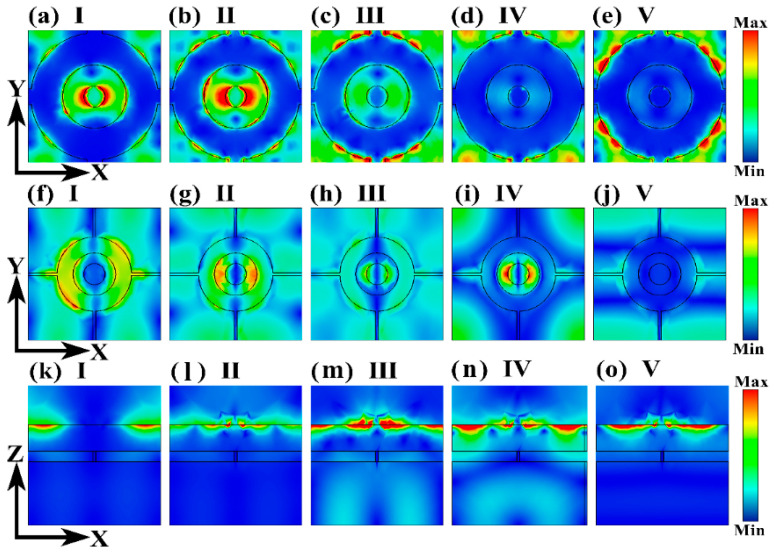
(**a**–**e**) show the electric field distribution on the XY plane of the graphene layer in the broadband absorber at the central frequencies of the five resonant absorption peaks. (**f**–**j**) illustrate the electric field distribution on the XY plane of the DSM layer at these same central frequencies. (**k**–**o**) depict the electric field distribution on the XZ plane at the interfaces of the absorber unit for the central frequencies of the five resonant absorption peaks.

To further investigate the physical mechanisms behind broadband absorption, we computed the electric field distribution at the boundaries of the graphene layer, DSM layer, and the absorber unit structure at the center frequencies of the five prominent resonant absorption peaks [52,53]. As illustrated in the figures, under the influence of the external electric field, LSPR excites the boundaries of both the graphene layer and the DSM layer. This localization of the electric field in these specific regions leads to significant energy loss of the incident electromagnetic waves, resulting in strong absorption [54]. A detailed analysis reveals a transition in the electric field resonance modes, as shown in Figure 4a–e. Specifically, the field patterns in Figure 4a,b are similar, with concentration around the inner and outer boundaries of the central graphene ring, while the field at the outer graphene boundary is notably weaker. Conversely, the field distributions in Figure 4d,e are similar, with the fields at the IV and V central frequencies concentrated at the boundary of the outer graphene, while the field at the graphene ring is very weak. The field pattern corresponding to Mode III represents an intermediate state, with the primary field concentration shifting from the graphene ring to the outer graphene boundary. This indicates that the broadband absorption achieved by the absorber results from the merging and expansion of adjacent resonance modes. Additionally, Figure 4f–j show that after the electromagnetic waves pass through the graphene layer and the upper dielectric plate to reach the DSM layer, different resonance modes are also excited. Figure 4k–o demonstrate that the electric field strength in the graphene layer is higher than in the DSM layer, indicating that graphene plays a predominant role in broadband absorption. The response modes of the DSM layer differ from those of the graphene layer, and its coupling with the graphene layer further enhances the absorber’s effectiveness in absorbing electromagnetic waves [55]. This explains the significant improvement in broadband absorption characteristics when combining graphene with a DSM compared to using a single graphene layer, as shown in Figure 2a.

**Figure 5:**
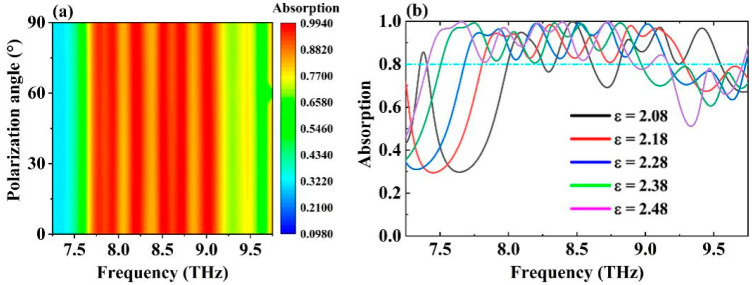
(**a**) The absorption spectrum of the wideband absorber in the range of 0–90° electric field polarization angle. (**b**) Absorption spectra of wideband absorbers under different dielectric constants of dielectric layers.

To explore the practical applicability of the absorber, we investigated its adaptability to different electromagnetic wave modes, as shown in Figure 5a. The absorber consistently exhibited excellent broadband absorption characteristics with high stability across a polarization angle range of 0–90°, demonstrating polarization insensitivity and suitability for complex electromagnetic environments [56,57,58]. Additionally, we examined the impact of variations in the dielectric constant of the absorber’s dielectric layer on the absorption spectrum, with the results presented in Figure 5b. As the dielectric constant of alumina increased from 2.08 to 2.48, a noticeable shift in the absorption spectrum occurred. At a dielectric constant of 2.08, the broadband absorption performance of the absorber significantly weakened, primarily due to changes in the dielectric constant affecting the relative impedance of the entire absorber, leading to poor matching with free space over a wide frequency range [59,60]. However, within the dielectric constant range of 2.18 to 2.48, the absorber maintained good broadband absorption characteristics, with a redshift in the central frequency from 8.548 THz to 8.16 THz, while the absorption bandwidth remained relatively stable. This variation in the dielectric constant enables effective modulation of the absorption performance. The redshift in the central frequency results from the dielectric constant’s effect on the relative impedance of the absorber, allowing it to achieve better impedance matching with free space at different frequencies.

**Figure 6:**
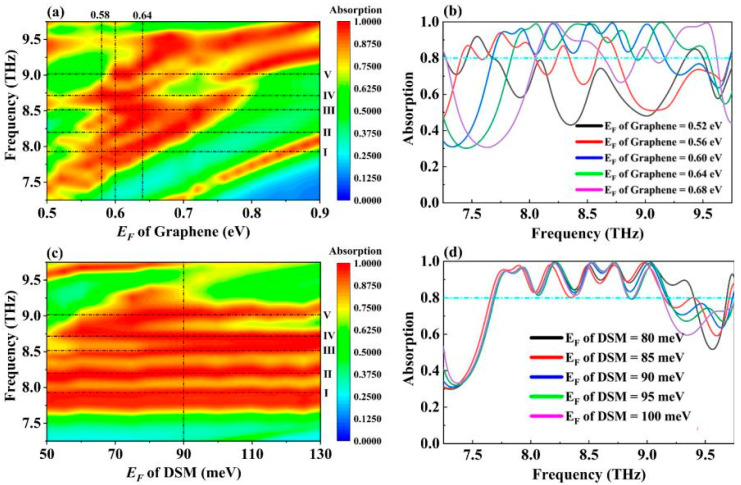
(**a**) Absorption spectra of wideband absorbers with Fermi levels of graphene in the range of 0.5–0.9 eV. (**b**) Changes in the absorption spectrum of the wideband absorber when the Fermi level of graphene varies in the range of 0.52–0.68 eV. (**c**) Absorption spectra of wideband absorbers at the Fermi level of DSM in the range of 50–130 meV. (**d**) Changes in the absorption spectra of wideband absorbers when the Fermi level of DSM varies in the range of 80–100 meV.

Given the dynamically tunable Fermi levels of both graphene and DSM, we employed a controlled variable method to separately investigate the relationship between the Fermi levels of these materials and the absorption spectrum of the absorber, exploring the graphene–Dirac semimetal dual-control adjustment mode [61,62]. When the Fermi level of the DSM was fixed at 90 meV, Figure 6a illustrates that as the Fermi level of graphene varied continuously within the range of 0.5–0.9 eV, the absorber’s absorption bandwidth exhibited a trend of initial increase followed by a decrease. Within the Fermi level range of 0.58–0.64 eV, the absorber displayed optimal broadband absorption characteristics with notable tunability of the absorption frequency. Specifically, at a graphene Fermi level of 0.58 eV, the absorber achieved a bandwidth of 1.364 THz with an absorption rate exceeding 80%, centered at 8.202 THz. As the graphene Fermi level increased to 0.64 eV, the absorption bandwidth expanded to 1.7 THz, with a blueshift of the central frequency to 8.694 THz, yielding a tunable range of 0.492 THz for the central frequency. The cause of this blueshift can be explained by the following equation:

(14) $$\lambda_{re}=a+bn_{sp}$$

In the equation, λ_sp_ represents the resonant wavelength, a and b are constants related to the geometric structure of the model and the surrounding dielectric constants, and n_sp_ denotes the effective refractive index of graphene. There is a negative correlation between the Fermi level of graphene and its effective refractive index. As the Fermi level increases, the effective refractive index decreases, leading to a blueshift in the resonant wavelength [63,64]. As depicted in Figure 6b, the average absorption rate of the absorber in the 7.25–9.75 THz range exhibited an initial increase followed by a decrease as the Fermi level of graphene rose from 0.52 eV to 0.68 eV. According to Equations (2) and (3), the conductivity of graphene increases with its Fermi level. This suggests that the surface plasmon resonance of the graphene layer first approaches saturation and then becomes oversaturated, which explains the observed trend in the absorption bandwidth [65,66].

Figure 6c,d illustrate the impact of varying the Fermi level of the DSM on the performance of the absorber when the Fermi level of graphene has been fixed at 0.6 eV [67]. Similarly to the variation in graphene’s Fermi level, an increase in the DSM’s Fermi level from 50 meV to 130 meV initially enlarged and then reduced the absorber’s absorption bandwidth, with a gradual splitting of Mode V. As shown in Figure 2, Mode V results from the combined effects of graphene and the Dirac semimetal, exhibiting the strongest coupling among all the resonant peaks. When the Fermi level of the DSM increased, the corresponding central frequency experienced the most significant blueshift. Overall, each of the five strong absorption peaks underwent a slight blueshift with increasing DSM Fermi levels, presenting an additional avenue for tuning the absorber’s performance. The absorber’s dual-control adjustment mode offers flexibility for practical applications, allowing for selection of the appropriate tuning method based on specific requirements.

**Figure 7:**
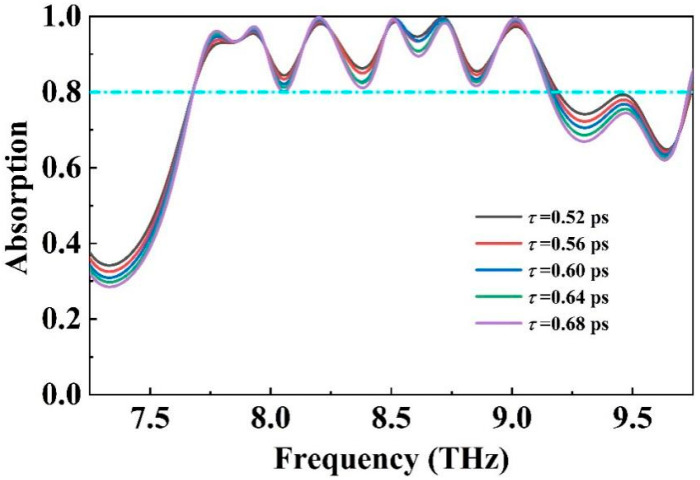
The relaxation time of graphene is increased from 0.52 ps to 0.68 ps in the absorption spectrum.

Moreover, as indicated by Equation (5), the incorporation of organic molecules on the surface of graphene in practical applications can effectively modulate its carrier mobility, thereby enabling control over the relaxation time without altering other parameters [68,69]. Figure 7 depicts the changes in the absorption spectrum of the absorber as the relaxation time of graphene increased from 0.52 ps to 0.68 ps. It is evident that while the absorption bandwidth and central frequency of the absorber remained relatively unchanged, there was a noticeable decline in absorption rate. This decrease is attributed to the fact that as the relaxation time increases, the carriers in graphene approach saturation, leading to reflection of the incident electromagnetic waves and thereby reducing effective absorption. This approach can be utilized to adjust the absorber’s absorption rate based on practical requirements.

## 4. Conclusions

In this study, the proposed broadband absorber achieved continuous broadband absorption with an absorption rate exceeding 80% within the 7.676–9.172 THz frequency range, exhibiting an absorption bandwidth of 1.496 THz and a central absorption frequency of 8.424 THz. The absorber features five prominent resonant absorption peaks within its broadband absorption range. Analysis of the absorber’s relative impedance reveals a good overall match with free space across the 7.676–9.172 THz band. The observed broadband strong absorption is attributed to the excitation of surface plasmons in both the graphene and DSM layers, with the coupling between these two metamaterials enhancing the fusion of adjacent resonant modes. Additionally, the absorber demonstrates excellent polarization insensitivity, maintaining robust broadband absorption performance under various incident electromagnetic wave modes. By varying the dielectric constant of the absorber’s dielectric layer, it is possible to tune the central frequency within the range of 8.51–8.548 THz while maintaining nearly unchanged absorption bandwidth and good performance, which is practically achievable. Furthermore, the dual-control tuning mode, enabled by adjusting the Fermi levels of the Dirac semimetal and graphene, allows for precise control over the absorption frequency. In practical applications, the choice of tuning method can be tailored to specific needs. Finally, while the relaxation time of graphene has a minimal impact on the absorption bandwidth and central frequency, it allows for adjustment of the absorption rate. In summary, the terahertz broadband absorber presented in this study leverages two distinct tunable materials to enhance flexibility and environmental adaptability. Its dual-control tuning mode suggests significant potential for applications in terahertz modulation and attenuation.

## Figures and Tables

**Table 1:** Structural parameters of graphene layer.

Parameters	Lx	Ly	Lz	h_1_	h_2_	h_3_	d	w	r_1_	r_2_	d_2_	w_2_	r_3_	r_4_
Unit (µm)	25	25	12	0.5	2	5	3	0.5	2	7	6	2.9	2	6

## Data Availability

Publicly available datasets were analyzed in this study. These data can be found here: [https://www.lumerical.com/] (accessed on 1 January 2020).
